# Soap Suds

**Published:** 1857-05

**Authors:** 


### SOAP SUDS

AT ten dollars a gallon! A money-making business that. But is any man so verdant as to pay such a price for an article which can be made for six cents a gallon? Yes, there are ten thousand men and women in New York city alone, who are regular customers, and have been for years in succession—at least so we judge from developments made at a special term of the Supreme Court, in the city of New York, for January, 1857, Judge Duer presiding. On the hearing, the receipt for making the "*Balm of a Thousand Flowers*" was produced, and it appeared that it was compounded of grease, lye, sugar, and alcohol, dignified with the name of palm oil, potash, &c. We have seen it recommended in the papers, with various certificates, as the best thing in the world to make the hair grow, to keep the face and hands clean, and to perfume the whole body generally. It so happens that it is a fact, that soap suds are the best thing known to keep people clean, to shave with, or to make the hair grow, when it can be made at all, or to keep it from falling out, when it has been brought to that state by plastering the scalp and hair with hogs' lard, or any other form of fat, for months in succession—this same oil being "*good for*" making all floating dust and dirt adhere to the hair, when, in a reasonable time, a layer of grease and dirt is found spread over the scalp, closing up the pores, destroying the vitality of the hair, causing it to fall out by the roots. Under such circumstances, the *Balm of a Thousand Flowers* is truly a useful article, for its thorough application will be followed by the growth of the hair, when it has been prevented from growing by accumulated filth, or by severe sickness. But then, soap suds will do the same thing, by adding a little spirits of hartshorn or alcohol. In our judgment, therefore, there is no *hair tonic* known more efficient and appropriate for the masses than a bottle of *Balm of a Thousand Flowers*, at one dollar, *or* half a pint of soap suds at one cent. ☞ Similar percentages do patent medicines yield, with the drawback however, of their failing uniformly to meet the reasonable expectations of the purchasers.

